# The effects of health-preserving sports on the treatment of COVID-19: A protocol for systematic review

**DOI:** 10.1097/MD.0000000000024201

**Published:** 2021-01-15

**Authors:** Yu Ji, Guorong Qiu, Dandan Song, Hezhi Liu, Li Chen

**Affiliations:** a Chengdu Sport University, Chengdu, Sichuan Province; b Chongqing University of Arts and Sciences, Chongqing; c Northwest Normal University, Lanzhou, Gansu Province, China.

**Keywords:** COVID-19, health-preserving sports, systematic review

## Abstract

**Background:**

From the end of 2019, COVID-19 has become a global epidemic, threatening the physical and mental health of everyone. How to effectively prevent and treat COVID-19 is concerned. Some studies have shown that Health-Preserving Sports plays an active role in the prognosis treatment of COVID-19. Therefore, this study aims to provide a method to assess the efficacy and safety of Health-Preserving Sports for the prognosis of COVID-19.

**Methods:**

This protocol is guided by the Preferred Reporting Items for Systematic Reviews. The following electronic databases will be searched: PubMed, the Cochrane Central Register of Controlled Trials, Excerpta Medica Database, MEDLINE, Web of Science, China National Knowledge Infrastructure Database, Chinese Biomedical Literature Database, China Science and Technology Journal Database, and Wan-Fang Database. We will be screened for data extraction and analysis, to summarize the therapeutic effect of Health-Preserving Sports on the treatment of COVID-19.

**Result:**

This study will provide a reliable evidence for the treatment of COVID-19 by Health-Preserving Sports.

**Conclusion:**

To provide a method to assess the efficacy and safety of Health-Preserving Sports for the prognosis of COVID-19, and guide future researches.

**PROSPERO registration number:**

CRD42020219526.

## 1 Background

In December 2019, some medical institutions have appeared unknown causes of pneumonia patients in Wuhan. Influenza and related diseases were monitored continuously in Wuhan, and 27 cases of viral pneumonia were found, all of which were diagnosed as viral pneumonia/pulmonary infection. December 31, 2019, the expert group of National Health Commission of the People's Republic of China arrived in Wuhan to carry out virus typing detection, isolation treatment, final disinfection, and so on. January 30, 2020, WHO recommends “2019-nCoV” as a temporary name for the virus (“n” = new disease, “CoV” = coronavirus), and declared the novel coronavirus outbreak a Public Health Emergency of International Concern (PHEIC), WHO's highest level of alarm. February 11, 2020, WHO announced that the disease caused by the novel coronavirus would be named COVID-19 (Corona Virus Disease 2019).^[1]^ March 11, 2020, COVID-19 identified by the WHO have pandemic characteristics. By November 19, 2020, globally have been 55,928,327 confirmed cases of COVID-19, including 1,344,003 deaths, reported to WHO.^[2]^

COVID-19 is an infection caused by the SARS-CoV-2 virus, SARS-CoV-2 is one of the coronaviruses, this virus was transmissible from person to person,^[3]^ people susceptible to SARS-COV-2 include of all ages.^[4]^ The mean incubation period is about 3 to 9 days, with a range between 0 and 24 days.^[5]^ infection can be spread by asymptomatic, presymptomatic, and symptomatic carriers,^[6]^ some studies have pointed out identified five potential transmission modes of COVID-19 including airborne, droplet, contact with contaminated surfaces, oral and fecal secretions,^[7]^ and attention to address the significance of aerosols with important implications for public health protection.^[8]^ The main clinical symptoms of COVID-19 patients include symptoms include fever, cough, fatigue, pneumonia, headache, diarrhea, hemoptysis, and dyspnea,^[9]^ some patients also report gastrointestinal symptoms such as diarrhea, vomiting, and abdominal pain.^[10]^ SARS-CoV-2 are widely acknowledged as severe traumatic events that impose threats not only because of physical concerns but also because of the psychological distress of infected patients.^[11]^

Exercise is known to mitigate many of the identified side effects from the pharmaceutical agents being trialled but has not yet been considered as part of management for COVID-19.^[12]^ In China, there are many Health-Preserving Sports as a common way for people to exercise, such as Six-Character Tactic, Tai Chi, Five-Animal Exercise, Eight-Section Brocade, and Muscle-Bone Strengthening. Tai Chi, which combines psychological treatment and physical exercise and requires no special equipment, is widely practiced in China and is becoming increasingly popular in the rest of the world.^[13]^ Five-Animal Exercise as the branch of traditional Chinese medicine, is a popular mind-body exercise in China and shown to improve emotional well being.^[14]^ A study present a modified version of rehabilitation exercises based on the underlying mechanism of the disease to mild cases of COVID-19, the modified rehabilitation exercises were retrieved from the Eight-Section Brocade, and are specifically designed for rehabilitation of COVID-19 patients at home or health facilities.^[15]^ However, there is no a systematic study to show the efficacy Health-Preserving Sports on COVID-19 patients. Therefore, this study aims to provide a method to assess the efficacy and safety of Health-Preserving Sports for the prognosis of COVID-19.

## 2 Methods

### 2.1 Registration

This systematic review protocol has been registered in International Prospective Register of Systematic Reviews (PROSPERO), registration number is CRD420202195260.

### 2.2 Inclusion criteria

#### 2.2.1 Study designs

We will include researches related to Health-Preserving Sports of patients suffering from COVID-19. Studies will be selected according to the criteria outlined below:

1. Published documents with complete data;
2. Participants were confirmed to have COVID-19 (positive nucleic acid test);
3. The intervention group received Health-Preserving Sports intervention for at least a period of treatment.
4. Include randomized controlled trials (RCTs), controlled (non-randomized) clinical trials (CCTs) or cluster trials, controlled before after (CBA) studies, prospective and retrospective comparative cohort studies, and case–control or nested case–control studies.

CBA studies will be included only if there are at least two intervention sites and two control sites. We will exclude cross-sectional studies, case series, and case reports.

#### 2.2.2 Participants

Patients diagnosed with COVID-19 of all ages and racial groups will be included, regardless of their sex, education level, or economic status. Postoperative infections, psychopaths, patients with severe pneumonia or other reasons who cannot exercise will not be included.

#### 2.2.3 Interventions

The studies at least one of the groups received Health-Preserving Sports intervention will be included, which can be carried out alone, or combined with other kinds of therapies. Health-Preserving Sports methods referred to in this article include Six-Character Tactic, Tai Chi, Five-Animal Exercise, Eight-Section Brocade and Muscle-Bone Strengthening Exercise. Excluding Boxing, Sanda, Mixed Martial Arts (MMA), and other Martial Arts Routines.

#### 2.2.4 Comparators

Comparisons will include retreats, drug therapy alone, and so on. In addition, the study will include studies that compare the use of Health-Preserving Sports in combination with another treatment vs Health-Preserving Sports alone, or that compare the use of Health-Preserving Sports in combination with another treatment versus the use of other treatments alone.

#### 2.2.5 Outcomes

Primary outcomes: The disappearance of the main symptoms (including fever, cough, Nucleic acid test, temperature recovery time) and indicators of body function (blood pressure, heart rate, body composition, muscle strength, range of motion of the joints, Activity of Daily Living), the return of white blood cell count to normal.

Secondary outcomes: The disappearance of the accompanying symptoms (such as myalgia, stuffiness, runny nose, headache, chest distress, nausea, vomiting, and diarrhea); the results of COVID-19 nucleic acid test are negative on 2 consecutive occasions (not on the same day), CT image improvement, and so on.

### 2.3 Data sources

The following electronic databases will be searched: PubMed, the Cochrane Central Register of Controlled Trials (CENTRAL), Excerpta Medica Database (EMBASE), MEDLINE, Web of Science, China National Knowledge Infrastructure Database (CNKI), Chinese Biomedical Literature Database, China Science and Technology Journal Database and Wan-Fang Database. Search dates: from inception dates to December 2020.

### 2.4 Data collection and analysis

#### 2.4.1 Search strategy

The search terms on PubMed are as follows: “Health-Preserving Sports” (and such as “Six-Character Tactic” OR “Tai Chi” OR “Five-Animal Exercise” OR “Eight-Section Brocade” OR “Muscle-Bone Strengthening Exercise”); “COVID-19” OR “Corona Virus Disease 2019” OR “Novel Corona Virus” OR “2019-nCoV”; “convalescence” OR “rehabilitation” OR “recovery”; “randomized controlled trial” OR “randomized” OR “randomly” OR “clinical trial.” According to the characteristics of the database, the comprehensive retrieval of the combined of Medical Subject Headings (MeSH) and text words was carried out. The full search strategy for PubMed is provided in Table 1, the same strategies are used in other electronic databases.

**Table 1:** Search strategy for the PubMed database.

Number	Search Items
1	Health-Preserving Sports
2	Six-Character Tactic
3	Tai Chi
4	Five-Animal Exercise
5	Eight-Section Brocade
6	Muscle-Bone Strengthening Exercise
7	1 or 2–6
8	COVID-19
9	Corona Virus Disease 2019
10	Novel Corona Virus
11	2019-nCoV
12	8 or 9–11
13	Convalescence
14	Rehabilitation
15	Recovery
16	13 or 14–15
17	Randomized controlled trial
18	Randomized
19	Randomly
20	Clinical trial
21	17 or 18–20
22	7 and 12 and 16 and 21

#### 2.4.2 Study selection

**Figure 1:**
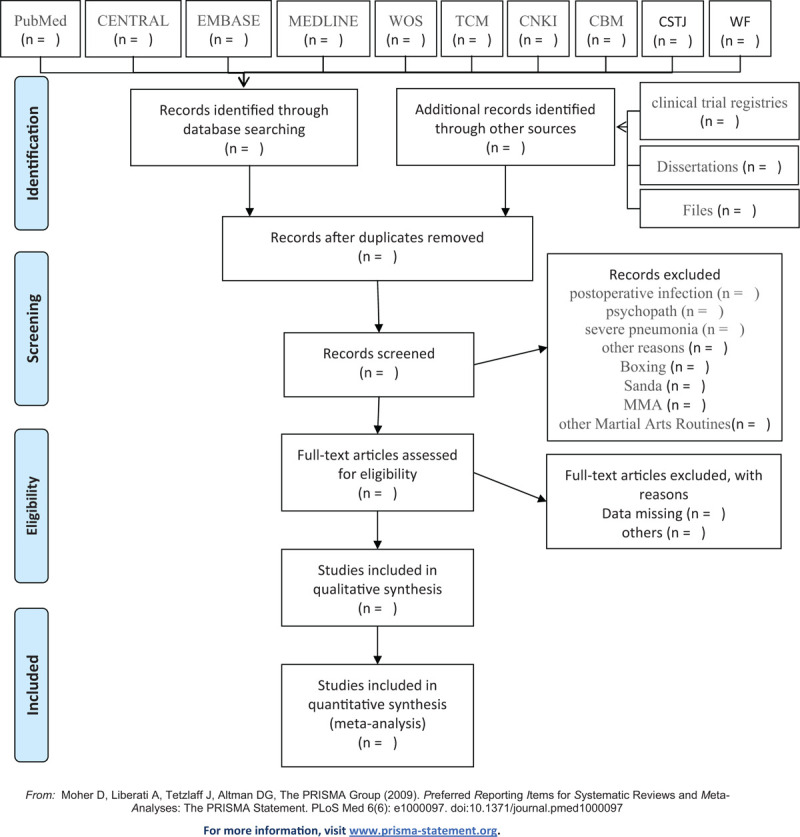
Flow chart of the study. Adapted from Preferred Reporting Items for Systematic Reviews and Meta-Analyses Protocols (PRISMA-P). CBM = Chinese Biomedical Literature Database, CENTRAL = Cochrane Central Register of Controlled Trials, CNKI = China National Knowledge Infrastructure, CSTJ = China Science and Technology Journal Database, EMBASE = Excerpta Medica Database, MMA = Mixed Martial Arts, TCM = traditional Chinese medicine, WF = Wan-Fang Database, WOS = Web of Science.

Before searching the literature, all reviewers will discuss and determine the screening criteria. After the screening requirements are clearly defined, the 2 reviewers will independently review and screen the titles and abstracts yielded by the search against the inclusion criteria, and then excluded some duplicate studies or studies with incomplete information. After literature retrieval, the literature records will be imported into EndNoteX9 software for management. Any inconsistency is resolved by discussing with the third reviewer. If the full text is not available, we will try to contact the appropriate author. We chose the PRISMA flow chart to show the process of selecting literature for the entire study (Fig. 1).

#### 2.4.3 Measures of effect

In this protocol, we will use 95% confidence interval (CI) risk ratio (RR) to rigorously analyze the dichotomous data. And for the continuous data, mean difference (MD) or standard MD (SMD) is used to measure the efficacy of 95% CI.

#### 2.4.4 Data extraction

The data will be extracted and recorded onto an Excel file, will include at least the following items: The title of the first author, publication journal name, year of publication, study sample size, intervention methods, intervention results, bias risk assessment, and findings. The result will be cross-checked by two reviewers, any disagreement will be resolved by consensus, and any persistent disagreement will be arbitrated by a third reviewer. We will contact the corresponding authors by telephone or email for additional information if necessary. All data will be analyzed by the Review Manager software (RevMan V.5.3).

#### 2.4.5 Risk of bias assessment

Two reviewers will use the Cochrane Handbook for Systematic Reviews of Interventions to assess the methodological quality of each trial. The risk of bias was evaluated for each study by random sequence generation, allocation concealment, blinding of participants and personnel, blinding of outcome assessment, incomplete outcome data, selective reporting, and other sources of bias. A judgment as to the possible risk of bias on each of the domains will be made from the extracted information; the risk of bias is evaluated at 3 levels: low risk, high risk, and unclear risk.

#### 2.4.6 Dealing with missing data

We will try our best to ensure the integrity of the data. When there are missing data, we will attempt to obtain missing data by contacting the corresponding author. If the corresponding author cannot be contacted, we will remove the experiment with incomplete data.

#### 2.4.7 Data synthesis

Each outcome will be calculated and combined using the RevMan 5.3. Specific implementation was based on the current version of the Cochrane Handbook for Systematic Reviews of Interventions. If tests of heterogeneity are not significant, the Mantel-Haenszel method will be chosen for fixed effect model, and if statistical heterogeneity is observed (*I*^2^ ≥ 50% or *P* < .1), the random effects model will be used. If heterogeneity is substantial, we will perform a narrative, qualitative summary.

#### 2.4.8 Subgroup analysis

If data are available, we will conduct a subgroup analysis according to different Patients characteristics, intervention method (Six-Character Tactic, Tai Chi, Five-Animal Exercise, Eight-Section Brocade, Muscle-Bone Strengthening Exercise), duration of treatment, and outcome measures.

#### 2.4.9 Sensitivity analysis

Sensitivity analysis is used to analyze research quality, intervention method, publishing type, and so on. The trials with quality defects will be excluded to ensure the stability of the analysis results.

#### 2.4.10 Grading the quality of evidence

We will use the evidence quality rating method to evaluate the results obtained from this analysis. Five factors: bias, inconsistent, inaccurate, indirect, and publication bias; 4 evaluation levels: high, medium, low, and very low.

### 2.5 Ethics and dissemination

The content of this article does not involve moral approval or ethical review because it is based on published literature. The results will be submitted to journal or conferences for publication and information sharing.

## 3 Discussion

This article mainly describes how to systematically review the therapeutic effect of Health-Preserving Sports on COVID-19, which mainly includes: Inclusion criteria, Data sources, Data collection, analysis, and so on. This study can provide some methods guidance for other scholars to study the prevention and treatment effect of Health-Preserving Sports on COVID-19 in the future. However, due to some limitations, also have shortcomings of this study.

## Author contributions

**Conceptualization:** Yu Ji.

**Data curation:** Dandan Song, Hezhi Liu, Li Chen.

**Formal analysis:** Yu Ji, Guorong Qiu, Dandan Song.

**Funding acquisition:** Yu Ji.

**Methodology:** Dandan Song.

**Software:** Guorong Qiu, Hezhi Liu, Li Chen.

**Writing – original draft:** Yu Ji.

**Writing – review & editing:** Guorong Qiu, Dandan Song.

## Abbreviations

Abbreviations: CBA = controlled before-after, CCT = controlled clinical trial, CI = confidence interval, COVID-19 = Corona Virus Disease 2019, MD = mean difference, MMA = mixed martial arts, PHEIC = Public Health Emergency of International Concern, RCT = randomized controlled trial, SARS-CoV-2 = severe acute respiratory syndrome corona virus 2, WHO = World Health Organization.
